# Inconsistencies and Controversies Surrounding the Amyloid Hypothesis of Alzheimer's Disease

**DOI:** 10.1186/s40478-014-0135-5

**Published:** 2014-09-18

**Authors:** Gary P Morris, Ian A Clark, Bryce Vissel

**Affiliations:** Garvan Institute of Medical Research, Neuroscience Department, Neurodegenerative Disorders Laboratory, 384 Victoria Street, Darlinghurst, NSW 2010 Australia; Faculty of Medicine, University of New South Wales, Sydney, Australia; Research School of Biology, Australian National University, Canberra, Australia

**Keywords:** Alzheimer's disease, Amyloid hypothesis, Amyloid beta, Neurofibrilliary tangles, Tau, Synapse, Microglia, Astrocytes, Neuroinflammation, TNF, Neurodegeneration, Amyloid precursor protein, Plaque

## Abstract

The amyloid hypothesis has driven drug development strategies for Alzheimer's disease for over 20 years. We review why accumulation of amyloid-beta (Aβ) oligomers is generally considered causal for synaptic loss and neurodegeneration in AD. We elaborate on and update arguments for and against the amyloid hypothesis with new data and interpretations, and consider why the amyloid hypothesis may be failing therapeutically. We note several unresolved issues in the field including the presence of Aβ deposition in cognitively normal individuals, the weak correlation between plaque load and cognition, questions regarding the biochemical nature, presence and role of Aβ oligomeric assemblies *in vivo*, the bias of pre-clinical AD models toward the amyloid hypothesis and the poorly explained pathological heterogeneity and comorbidities associated with AD. We also illustrate how extensive data cited in support of the amyloid hypothesis, including genetic links to disease, can be interpreted independently of a role for Aβ in AD. We conclude it is essential to expand our view of pathogenesis beyond Aβ and tau pathology and suggest several future directions for AD research, which we argue will be critical to understanding AD pathogenesis.

## Introduction

*“Whenever a theory appears to you as the only possible one, take this as a sign that you have neither understood the theory nor the problem which it was intended to solve.”*

- *Karl Popper*

A hypothesis that remains unproven yet catches the collective imagination can become, with the passage of time, so seductive that it dominates peer review opinion and arrests the development of alternative ideas. Such is the case for the amyloid hypothesis of AD. From the mid-1980s [1,2] this hypothesis began to give focus and excitement to what had been an unstructured research field with dozens of complex and unrelated theories [3], none of which dominated. It became a simple and effective way to describe AD pathogenesis to funding bodies, pharmacological companies, and the public at large.

The hypothesis arose through the input of researchers with a history of observing prion particles [4,5] seeing parallels between these entities in brain sections in Creutzfeld-Jacob disease and the plaques in AD brain, described years earlier [6]. It warrants recalling that a commentary [7] notes Alzheimer devoting only two sentences of his 1907 text to these plaques, and there being no reason to suppose that he or indeed anyone until the early 1980s, saw them as causal.

When Prusiner and Master's interest in these plaques began, others showed they consisted of a novel amyloid fibril [1,8] containing highly aggregating small polypeptides about 40 amino acids long with a molecular mass of 4kDa, now known as amyloid-beta (Aβ) [9]. The dense fibre-like tangles Alzheimer noted, now termed neurofibrilliary tangles (NFTs), contain bundles of paired helical filaments of the microtubule associated protein tau [10]. The 1980s ended with a report that the Aβ peptide derived from the amyloid precursor protein (APP) was neurotoxic [11], transforming a histological parallel into the amyloid theory of disease pathogenesis.

**Figure 1:**
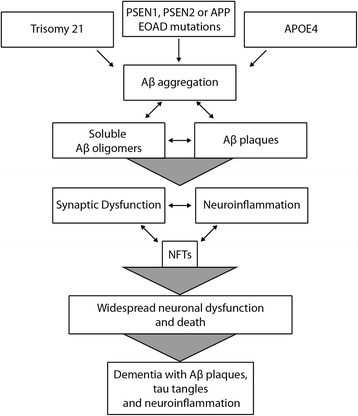
**The Amyloid Hypothesis.** The amyloid hypothesis postulates that Aβ aggregation triggers a cascade of events ultimately resulting in AD. Familial mutations in *PSEN1*, *PSEN2* or *APP* are associated with early-onset AD (EOAD). These genetic risk factors are postulated to impact the cleavage of Aβ from APP, leading to oligomerisation and eventual Aβ plaque formation. Individuals with trisomy 21 (Down’s Syndrome), and therefore a triple copy of *APP*, suffer EOAD. The strongest genetic risk factor for late-onset AD (LOAD) is the presence of at least one *APOE4* allele. It is unclear as to what triggers Aβ accumulation in LOAD, though it is suggested that there may be a number of contributing factors such as reduced Aβ clearance due to *APOE* genotype. Aβ oligomerisation is proposed to trigger a cascade involving the formation of neurofibrilliary tangles (NFTs) composed of hyperphosphorylated tau, synapse loss, neuron death and widespread neuroinflammation, particularly in brain regions involved in learning and memory, such as the hippocampus. As the amyloid burden increases, the ongoing catastrophic loss of synapses and neurons is thought to lead to progressive dementia.

Hence the basis of AD became, in essence, Aβ killing neurons, and later also Aβ killing synapses, despite the syndrome clearly being more subtle and complex and the fact that histopathology lesions have a poor record of being causal in disease pathogenesis. Although the amyloid hypothesis has shifted its focus from plaque to soluble forms of Aβ, it largely remains defined by the central tenet that accumulation of amyloid, in a variety of forms, triggers a cascade that harms neurons and synapses (Figure 1).

The amyloid hypothesis has become difficult to challenge because it is so often the lens through which peer reviewers, granting bodies and pharmaceutical companies view, judge and support AD research. Thus new non-amyloid data tends to be couched in terms that place it within the amyloid hypothesis and many authors tacitly ignore valid, but quite different, interpretations.

We show here however that the central conclusion of the amyloid hypothesis, that Aβ is the cause of AD is, at very least, premature. Aβ is one product of amyloid precursor protein (APP) processing. Current data does support a conclusion that aberrant expression and processing of APP may sometimes cause human familial AD (FAD), also called early-onset AD (EOAD), and that Aβ, in excess, can be toxic. However, data does not support a conclusion that aberrant Aβ expression is the cause of sporadic AD, also known as late-onset AD (LOAD). In fact as we show data suggests that aberrant Aβ expression may not be the primary cause of all EOAD. Instead, it may more often play a role, perhaps secondary, as part of more complex processes in the CNS. We suggest the field has matured sufficiently, with a range of alternative interpretations available, that a strong prospect for a change in direction exists that could provide a major advance in disease understanding and clinical interventions.

## The Amyloid Hypothesis

The amyloid hypothesis postulates that amyloid-beta (Aβ), in a variety of forms, triggers a cascade harming synapses and ultimately neurons, producing the pathological presentations of Aβ plaques, tau tangles, synapse loss and neurodegeneration, leading to dementia. Aβ accumulation is thought to initiate AD pathology by destroying synapses, causing formation of NFTs, and subsequently inducing neuron loss (Figure 1). Although some changes to the hypothesis have occurred since the original publications, notably a shift toward defining soluble Aβ oligomers as the toxic agent, rather than plaques, the theory and the way data is interpreted have remained largely the same, i.e. Aβ accumulation as oligomers or plaques triggers AD. A large, growing literature espouses the amyloid hypothesis. In this section we summarise these data and how the dominance of this hypothesis arose.

### Putative evidence in support of the hypothesis

Using the amino acid sequence corresponding to Aβ [9], the major constituent of amyloid plaques in AD, a precursor gene cDNA to Aβ (the *amyloid precursor protei*n, *APP*) was sequenced and mapped to chromosome 21 [12]. This finding had compelling implications in view of the observation many individuals with trisomy 21 (Down’s Syndrome) reach the neuropathogical criteria for AD by age 40 [13]. Such Down’s individuals would have a triple copy of *APP*, and therefore it was reasoned, excess Aβ production. Since Aβ is the main component of plaques seen in AD, it is presumed in turn that excess Aβ is the cause of AD in Down’s syndrome. Surprisingly, the fact that not all people with Down’s syndrome develop AD, despite plaques and increased Aβ expression, did not receive significant attention [13]. This observation may have quelled consideration of Aβ as the sole risk factor for AD.

**Figure 2:**
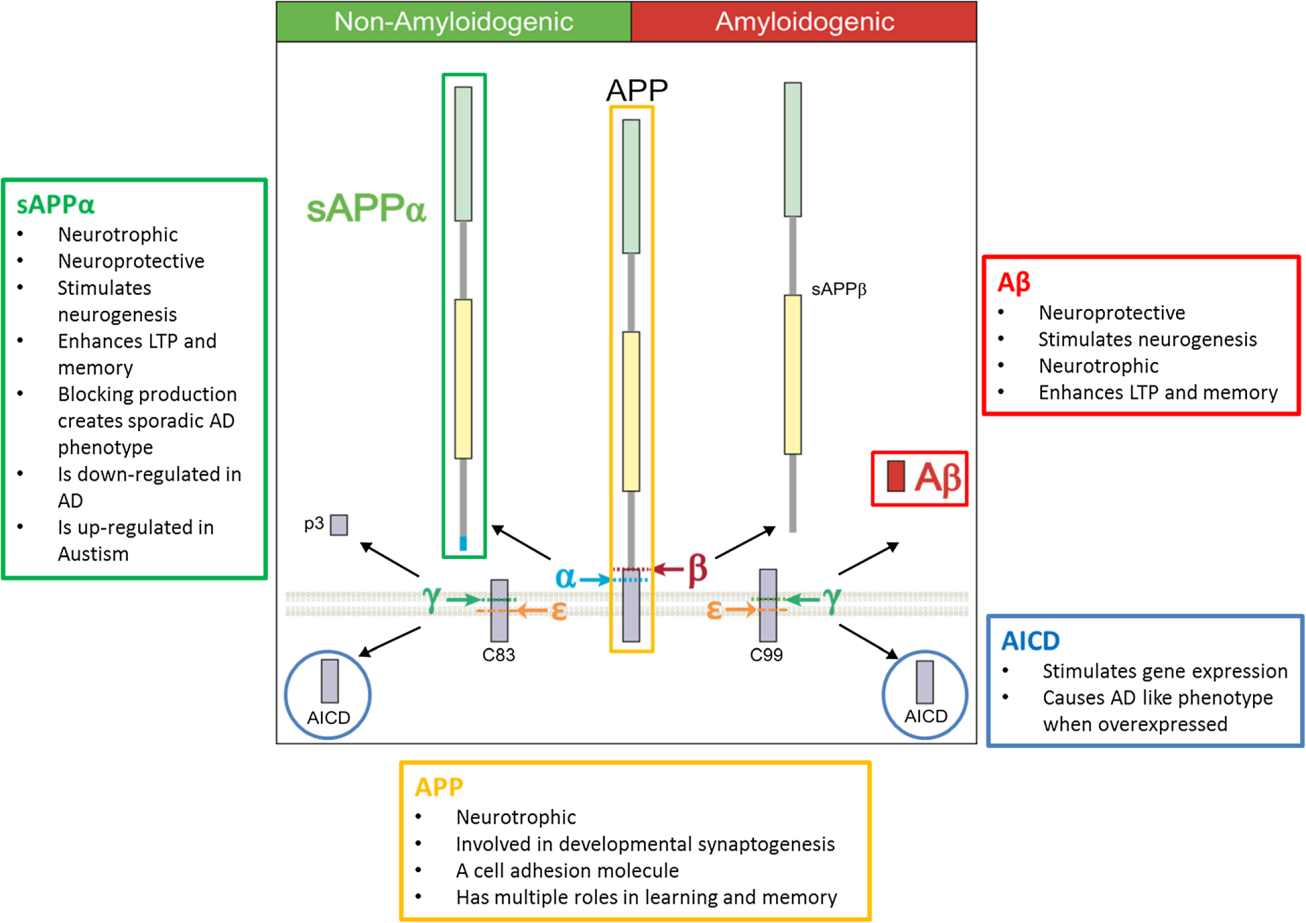
**Cleavage of APP and Physiological roles of APP and APP Fragments.** Amyloid precursor protein (APP) can be cleaved via two mutually exclusive pathways. Importantly, various studies have suggested that these various fragments of APP processing, including Aβ, can have a number of possible roles in normal brain physiology, shown in the boxes. In the so-called amyloidogenic pathway APP is cleaved by β-secretase (beta-site APP cleaving enzyme 1 (BACE1)) and γ-secretase enzymes (PSEN1 is the catalytic core of the multiprotein γ-secretase complex). The initial β-secretase cleavage produces a large soluble extracellular domain, secreted amyloid precursor protein-β (sAPPβ). The remaining membrane bound C99 stud is then cleaved by multiple sequential γ-secretase cleavages. These begin near the inner membrane at a γ-secretase cleavage site epsilion (the ε-site) to produce the APP intracellular domain (AICD), and then subsequent sequential γ-secretase cleavages trim the remaining membrane bound component to produce different length Aβ peptides including Aβ43, Aβ42, Aβ40 and Aβ38 [17]. In the so-called non-amyloidogenic pathway APP is processed consecutively by α- and γ-secretases to produce secreted amyloid precursor protein α (sAPPα), p3 (which is in effect Aβ17-40/42) and AICD. The major α-secretase enzyme is A Disintegrin and metalloproteinase domain-containing protein 10 (ADAM10). Cleavage via amyloidogenic and non-amyloidogenic pathways depends on the cellular localisation of cleavage enzymes, and of full-length APP, which are expressed and trafficked in specific sub-cellular locations.

Next, studies of familial EOAD uncovered genetic links between the *APP* gene and AD [14]. APP is processed into smaller peptide fragments, one of which is Aβ, via cleavage by α-, β- and γ-secretases (Figure 2). Importantly, EOAD-linked point mutations were identified not only in *APP* itself but also in *presenilin-1* (*PSEN1*) and *presenilin-2* (*PSEN2*) [15,16] the key catalytic subunits of γ-secretase, known to cleave APP (Figure 2). No known AD-causing mutations are present in the gene encoding the β-secretase gene, *beta-site APP cleaving enzyme 1* (*BACE1*).

The genetic mutations are reasoned to cause AD through aberrant processing of APP, leading to either increased levels of Aβ or an increased production of the 42 and 43 amino acid forms of Aβ (Aβ42/Aβ43) over the 40 amino acid form of Aβ (Aβ40). It is argued this triggers aggregation of Aβ [17]. The discovery that transgenic mice expressing familial human *APP* and *PSEN* mutations recapitulate many, but not all, of the features of the human disease [18] further established the link between aberrant Aβ production and the AD phenotype. This latter discovery, perhaps more than any other, tied the field to the amyloid hypothesis for the next decades.

The conclusions of the aforementioned studies were grounded in an unquestioned assumption that Aβ, rather than altered expression of APP or its products, causes AD pathology. The assumption arose because Aβ was the key component of plaques and because Aβ caused neurotoxicity in healthy cells [19]. Further, hyperphosphorylation of tau, thought to be downstream of Aβ, was seen as a critical mediator of the neurotoxic effects of Aβ [20] placing Aβ at the top of the pathological chain of AD events. A cycle thus began to develop early whereby studies were designed and then interpreted on the basis of the hypothesis that Aβ caused AD pathology, rather than being critically evaluated in the context of a range of possible interpretations.

Further, given the impact discoveries of mutations in *APP*, *PSEN1* and *PSEN2* have had in driving the amyloid theory, it is notable that, while these mutations account for the majority of EOAD cases, EOAD only comprises less than 5% of all AD cases [21]. In fact, the majority of AD cases are sporadic, idiopathic LOAD. It seems in retrospect presumptive to have extrapolated a role for Aβ in all AD based on the genetic evidence suggesting a role for altered *APP* processing in EOAD.

In general, the risk genes identified for LOAD are subtle, with no direct genetic association to the *APP* gene or its processing enzymes. The most well-known genetic link to LOAD is the *apolipoprotein* genotype E4 (*APOE4*) [22]. Recently another strong risk gene for LOAD was identified, a variant of the *triggering receptor expressed on myeloid cells 2* gene (*TREM2*), implicating excessive innate immunity in Alzheimer’s pathogenesis [23]. Although these two mutations have been the strongest to date, many more have been associated with LOAD. Most of these genetic risk factors have been interpreted through the lens of the amyloid hypothesis, mainly by considering their modulatory effects on Aβ, though other interpretations are equally valid, an issue we discuss further in later sections.

### The crucial role of synapse loss in AD

Synapse loss leads to a loss of dendritic mass [24] and, crucially, may precede, and indeed drive, neuron loss in a range of conditions [25]. Deficits in synaptic plasticity are measurable at just one month of age in mouse models of AD [26] and synapse loss is evident during early stages of the human disease [27]. Elegant research has revealed the number of neocortical synapses to be a better correlate of cognition than both Aβ plaques and NFTs [28] and a greater loss of synapses than neurons is evident in human AD brains [29]. These observations place synapses at the forefront of understanding AD pathogenesis. It has therefore been suggested that AD is primarily a synaptic disorder [30,31]. In a mouse model of AD, synapse loss arises from over-elimination of synapses, rather than a failure of synapse formation [32].

#### Putative evidence that Aβ causes synapse dysfunction and loss *in vitro* and *ex vivo*

The obvious caveat of *in vitro* studies is that they may not represent actual processes in the brain. Nevertheless, much work focuses on the impact of Aβ oligomers on synapses, most of it in *in vitro* or *ex vivo* culture systems.

Key *in vitro* findings are summarised as follows; Aβ oligomers bind exclusively and rapidly to synaptic terminals [33], altering both pre- and postsynaptic structures in cultured neurons and affecting excitatory, but not inhibitory nerve terminals [34]. The effects of Aβ on synapse formation, neurite outgrowth and arborisation is concentration-dependent [35] and rapidly decreases expression of memory related receptors such as NMDA and EphB2 [33].

In *ex vivo* organotypic slices; physiological concentrations of Aβ dimers and trimers, but arguably not monomers, induce loss of hippocampal synapses, which requires the activation of NMDARs [36]. Sub-lethal levels of Aβ decrease spine density, increase spine length and subdue spine motility [37]. Selective expression of APP in pre- or postsynaptic neurons, resulting in either dendritic or axonal Aβ overproduction, reduces spine density and plasticity at nearby dendrites [38]. Some molecular mechanisms of Aβ-induced synaptic dysfunction and spine shrinkage in these *in vitro* and *ex vivo* paradigms have been suggested [39].

#### Putative evidence that Aβ can lead directly to synapse dysfunction *in vivo*

Despite several technical limitations, arguably the most direct evidence supporting the role of Aβ in synapse destruction in AD is that Aβ oligomers extracted from human AD brain inhibit long-term potentiation (LTP), enhance long-term depression (LTD), reduce dendritic spine density and disrupt memory and learning *in vivo* when directly injected into a mouse hippocampus [40]. Hippocampal injections of soluble Aβ42 oligomers *in vivo,* in awake mice, stimulate AD pathology including neuronal loss, although this requires a regimen involving multiple injections of highly concentrated Aβ [41]. Finally, transgenic mouse lines producing high levels of soluble Aβ show age- and genotype dependent reductions in spine density [42].

### Is Aβ the central cause of synapse destruction in AD?

The aforementioned studies of Aβ at synapses are difficult to interpret. Aβ plaque deposition can occur without associated synapse loss [43], and conversely synapse and dendritic tree loss can occur in areas without Aβ deposition, although synapse loss does usually appear exacerbated near Aβ plaques [44]. Thus it would be prudent to treat the suggestion that Aβ plaques have a primary causative role in synapse destruction in human LOAD with caution.

As for Aβ oligomers however, while many reports identifying Aβ oligomers as triggers of synaptic degeneration support the amyloid hypothesis, technical restrictions limit interpretations of these results, and their relevance to the human disease is unclear, as we discuss below. For example, are pathological effects in mice resulting from injection or over-expression of Aβ relevant to the human condition?

Furthermore, synaptic gene dysregulation in early AD can occur independently of alterations in the expression of APP and regulators of APP metabolism [45]. Finally, genetic studies suggesting a role for APP and its processing in familial EOAD may have been incorrectly extrapolated to LOAD. Thus, as with plaques, it is conceivable that Aβ oligomers play a role, but it would also be prudent to treat the suggestion Aβ oligomers play a primary or sole causative role in synapse destruction with a degree of caution.

Later we discuss our view that understanding AD requires first understanding the complex biology of the multicellular synapses [46], the role of glia in synapse removal, and the means by which these cells can be driven towards excess synapse removal and/or destruction.

## The Amyloid Hypothesis and Recent Drug Developments

Since the literature on AD has been largely Aβ-centric, myriad studies provide much reassurance that the amyloid hypothesis is on solid ground. As a result, the hypothesis has maintained supremacy in driving drug development efforts.

Much faith has been placed in AD mouse models, built on and embedded in the amyloid hypothesis, as the testing ground for new therapies. Beginning with a 1999 study by Schenk and colleagues [47], many studies show that amyloid removal relieves AD symptoms in mouse models of the disease. Since these mice produce human amyloid, both active and passive immunization strategies aimed at removing the putative causal Aβ, not surprisingly, reduce fibrillar amyloid and Aβ plaque deposition, result in fewer neuritic lesions, and protect mice from cognitive decline [48]. Furthermore, inhibitors of the enzymes that cleave Aβ from its membrane bound precursor have been therapeutically investigated both in mice and human studies (Table 1). Such positive outcomes rapidly led to Phase 1, 2 and 3 human trials.

**Table 1:** **High profile clinical trials based on the amyloid hypothesis**

**Mechanism of action**	**Drug name**	**Clinical phase**	**Key results from each trial**	**Current status (August 2014)**	**Reference**
**Active immunisation with Aβ**	AN1792	2	Plaque Cleared. NFT reduced in neuronal processes, but not cell bodies. Very few antibody responders (25/239). Reports of encephalitis.	Discontinued	[49,50]
	CAD106	2	Favourable safety profile. Prolonged antibody titre in responders.	Ongoing	[51]
	ACC001	2	Co-administration of adjuvant required for strong antibody response. Generally safe and well-tolerated, no adverse related event.	Discontinued	[52]
	AD02	2	Favourable safety and tolerability profile. Did not reach primary or secondary outcome measures in phase 2.	Ongoing	[53]
**Passive immunization against Aβ**	Solanezumab	3	Worsening cognition compared to placebo, multiple adverse events.	Terminated	[54]
	Bapinezmab	3	Engaged target. Reduction in cerebrospinal fluid phospho-tau in APOE4 carriers. Decreased rate of amyloid accumulation in APOE4 carriers. No improvement in clinical outcomes in carrier or non-carriers of APOE4. Negative amyloid scans in 36% of non-carriers.	Discontinued	[55]
	Gantenerumab	2/3	Safe and well-tolerated at phase 1. Focal inflammation in areas with amyloid reduction a concern. Amyloid reductions compared to placebo.	Recruiting for Phase 3 DIAN trial	[56]
	Crenezumab	2	Did not meet co-primary endpoints. Trend of improved cognition in people with mild disease.	Ongoing	[57]
	Ponezumab	2	Safe and well-tolerated at phase 1. Plasma Aβ40 increased at phase 2. No effect on primary endpoints in phase 2.	Recruiting for further Phase 2 trials	[58]
**γ-Secretase inhibitors**	Avagacestat	2	Gastrointestinal and dermatological side effects at Phase 1. Also dose-dependent pharmacodynamic effects on CSF biomarkers in some patients. Trend towards worsening cognition at higher doses compared to placebo. Amyloid related imaging abnormalities.	Discontinued	[59]
	Semagacestat	3	Dose-dependent reduction in Aβ synthesis at Phase 1. Reduced plasma Aβ at Phase 2, but no differences in cognition. No improvement in cognition and worsening cognition at higher doses compared to controls at Phase 3.	Discontinued	[60]
**γ-Secretase modulators**	CHF5074	2	Anti-inflammatory at Phase 2. Trend towards improved function in APOE4 carriers.	Ongoing	[61]
	EVP-0962	2	Does not inhibit cleavage of γ-secretase substrates other than APP.	Ongoing	[62]
	Tarenflurbil	3	Small functional benefit at higher doses in mild AD but no cognitive benefit at Phase 2. No changes in CSF Aβ42. Failed to meet primary and secondary endpoints at phase 3.	Discontinued	[63]
**β-Secretase modulators**	MK-8931	3	Reduced CSF Aβ compared to controls. Safe and tolerable at Phase 2.	Recruiting for Phase 3	[64]
	CTS-21166	1	Dose dependent reduction in plasma Aβ.	Completed	[65]

### The amyloid hypothesis has so far failed clinically

The Food and Drug Administration (FDA) has over the years approved five drugs for AD; Donepzil, Galantamine, Memantine, Rivastigimine and Tacrine. It is notable that each of these are unrelated to the amyloid hypothesis and were not tested in transgenic AD mice before being used in the clinic [66].

Meanwhile, many anti-amyloid treatments that were tested in mice have completed, or are undergoing, extensive clinical trials in humans. We summarise the most high profile of these drugs in Table 1. They are divided into those directly targeting Aβ by active and passive immunization, those targeting inhibition or modulation of the γ-secretase APP cleaving enzyme (Figure 2), presenilin, and those targeting the APP β-secretase cleavage enzyme BACE1.

So far, anti-Aβ treatments have broadly failed to meet their primary clinical endpoints and some major phase 3 trials were halted early. None of the tested treatments have produced a discernible functional recovery, or altered the course of disease. In fact alarmingly some, specifically inhibitors of γ-secretase, lead to an increased decline in cognition (Table 1). With each successive failure the validity and foundations of the amyloid hypothesis, on which these drugs have been based, is called increasingly into question. Haste to run Phase 3 trials without Phase 2 success, and similar criticisms, have recently been made of this commercially-driven enterprise [67].

Why is the hypothesis failing clinically? Some suggest the disease is not being targeted early enough [68], noting that in animal models anti-Aβ approaches clear hyperphosphorylated tau aggregates when given to young, but not old, animals [69] and, also, detailed analysis of recent trials have shown hints of treatment benefit in individuals treated early in disease [57].

Planned human intervention studies aim to address this issue in two ways. The DIAN [70] and the API Colombia study [71] use anti-Aβ antibody treatments in presymptomatic individuals at risk for familial EOAD. If these trials succeed, the results will provide evidence for a degree of Aβ involvement in EOAD. They will not necessarily prove Aβ causality in all EOAD, nor will they provide information on the role of Aβ in LOAD.

The Anti-Aβ asymptomatic Alzheimer’s disease trial [72] meanwhile tests the effect of starting anti-Aβ treatment at the pre-symptomatic stage, in individuals predicted to develop LOAD on the basis of brain amyloid accumulation as measured by positron emission tomography (PET) imaging. This will effectively test the hypothesis that anti-Aβ treatments provide cognitive benefits when given earlier in sporadic AD.

Another prominent suggested reason for clinical failures of anti-Aβ drugs in particular are that the agents used initially were not properly validated and were flawed [68]. A recent study has shown the monoclonal anti-Aβ antibodies, solanezumab and crenezumab, fail to target human Aβ as effectively as they target over-expressed human Aβ in mouse models [73]. The possibility was also countenanced that only amyloid plaques, potentially functionally inert [74], rather than soluble Aβ oligomers were targeted in early trials. Furthermore monotherapies may not be capable of effectively reducing Aβ plaque load. A double pronged approach to reduce Aβ by both active immunisation and inhibition of β-secretase has effectively cleared plaques in mice [75]. However, as reviewed recently [67], therapeutic approaches targeting plaque and approaches targeting soluble Aβ have both now been tested in humans, with equally negative outcomes.

Whilst the latter conclusions suggest that anti-Aβ treatments may be failing because they poorly target Aβ in human tissue, the conclusion does not disprove an alternative view for the failure of clinical trials, namely that Aβ is not responsible for all AD. Indeed for some the failure of clinical trials was by no means a surprise [76] and the validation that bapineuzumab does effectively bind Aβ in human tissue [73], but did not provide recovery in clinical trials, only provides support for this view. In the following sections we elaborate on the notion the central focus on Aβ is, on the available evidence, unwarranted.

## Evidence Supporting the Amyloid Hypothesis is Equivocal

### The Aβ deposition paradox

*“How wonderful that we have met with a paradox. Now we have some hope of making progress.”*

- Niels Bohr

#### Aβ deposition occurs in cognitively normal individuals

Up to 40% of non-demented elderly can reach some level of neuropathological criteria for AD [77]. A positive correlation also exists between Aβ deposits and increases in phosphorylated tau, the other major cerebral histological inclusion in AD protein, in cognitively normal patients [78]. In one study only 17% of cognitively normal elderly patients had few or no degenerative brain changes [79], and in neuroimaging amyloid-PET studies 10-30% of cognitively normal individuals have amyloid-positive scans [80]. Around 50% of people over the age of 85 have AD [81], rising to 77.5% of centenarians who meet criteria for mild confusion or severe dementia based on cognitive testing [82]. This could be interpreted to suggest that amyloid deposition is predominantly associated with normal aging and is not a disease *per se*.

Not only does this paradox create difficulty for diagnosing disease by Aβ plaque deposition, it remains awkward for the amyloid hypothesis. It is suggested that individuals with high plaque burden, but are cognitively normal, are in a pre-clinical AD stage [83], since the progression of mild-cognitive impairment to AD is associated with the Aβ deposits [84]. However recent *in vivo* imaging techniques illustrate that some non-demented patients can have plaque burdens equivalent to those seen in demented patients [85], and amyloid deposition commonly plateaus, despite declining cognition [86]. In contrast, other markers of advancing AD pathogenesis such as synaptic loss, NFTs, and microglial activation correlate with the course of disease [87]. Conversely, neurodegeneration can appear independently of plaque deposition [88]. Notably too, individuals with Trisomy 21 (Down’s syndrome), who have a triple copy of *APP* universally have elevated Aβ and diffuse non-fibrillar plaques that begin developing as early as 8 years of age, yet they do not necessarily develop dementia by their 70s [13]. Thus, the link between Aβ deposits and causality remains uncertain.

In sum, the distribution of amyloid deposits in the brain does not correlate well with neuropathology, loss of neural function from specific brain areas, or cognitive impairment. A conclusion that plaque is not the cause of LOAD provides one possible explanation for Aβ vaccination trials not improving patient outcome, even when plaque was removed.

#### Why are plaques present in cognitively normal individuals?

Several valid interpretations of AD data could equally explain the Aβ deposition paradox:

1. The type of plaque is important for cognitive decline. Plaques can be either diffuse, fibrillar or dense cored, and fibrillar amyloid plaques may represent the toxic plaque in the AD brain [89]. Some suggest a rise in fibrillar plaque load is correlated to dementia [90], but both diffuse and fibrillar plaques exist in cognitively normal people [91] and in any event plaques are questioned as a cause of cognitive deficits [92].
2. Plaques may be non-toxic, but could become toxic when bound to metal ions [93].
3. Some individuals may have a ‘cognitive reserve’ , a hypothetical concept described as accumulating over a lifespan, allowing them to cope with more amyloid [94].
4. Amyloid fibrils may be biologically inert [74] casting doubt over the role of these lesions in the AD brain.
5. Amyloid plaques are not the cause of AD, rather it is soluble Aβ oligomers, a theory with limitations (discussed in detail below).
6. Another possibility, difficult to resolve, is that Aβ plaque load contributes in only some cases of AD, together with the simply corollary that it has little to do with outcome in many cases of the diseases.
7. Plaques could be an occasional by-product of APP cleavage with variable, if any, mechanistic consequence.
8. Plaques may be formed for a purpose, as a cerebral blood vessel sealant to maintain vascular supply to the brain during aging [95]. The implications of reduced clearance of brain Aβ and the presence of amyloid plaques in the cerebral vasculature are reviewed in depth elsewhere [96].

Causal or not, why do Aβ plaques accumulate? Studies of EOAD mutations suggest they arise from increased cleavage of longer, more amyloidogenic forms of Aβ. However, this does not explain Aβ plaques in LOAD cases lacking EOAD mutations, and in non-demented individuals. Failed clearance of Aβ via reduced levels of Aβ cleaving enzymes such as neprilysin [97] and insulin-degrading enzyme [98] may allow plaque to accumulate. Also, the risk allele *APOE4* may relate to reduced Aβ clearance from the brain [99]. Whilst used as evidence for the amyloid hypothesis, these latter explanations for plaque accumulation also fit with the other plausible explanations for plaque accumulation outlined above.

#### The rise of the soluble Aβ hypothesis

Rather than considering that the unreliability of plaque as a disease marker may reflect badly on the amyloid hypothesis, its guardians embraced soluble Aβ oligomers as a cause of AD [100]. Yet, as we elaborate in the next section, concerns have been voiced about the oligomer hypothesis. These include a suggestion the amyloid hypothesis is “*…that invisible molecules target invisible structures*” [101], with new information interpreted within a constantly fluctuating amyloid hypothesis, rather than being molded into alternative hypothesis which may better explain disease causality. Regardless, the amyloid hypothesis has shifted in recent years to suggest soluble Aβ oligomers, rather than plaques, are responsible for neurodegeneration.

### Uncertainty surrounding the presence of Aβ oligomers *in vivo*

Oligomers are seen in the brain tissue of AD mouse models, although this does not correlate with cognitive decline [102]. Nevertheless, in one study oligomers were found in post-mortem human AD brains but not cognitively normal controls [40]. Furthermore, oligomers appeared to differentiate AD, dementia without AD pathology and cognitively normal patients in another [103]. This evidence is much cited in support of a role for oligomers in AD, but the data fail to define clearly which manifestation of many possible Aβ oligomers are toxic, or if Aβ oligomers are responsible for toxicity [104].

Debate also continues over the nature of amyloid assemblies *in vivo*, with studies reporting various assemblies showing different toxic effects (reviewed in [105]). Moreover, at present it is only possible to study Aβ oligomers secreted from *in vitro* cultures, or extracted from post-mortem brain tissue [105,106]. Accordingly, some have inferred current evidence for Aβ oligomerisation may simply be an artifactual consequence of detection techniques such as sodium dodecyl sulphate (SDS) polyacrylamide gel electrophoresis (PAGE) [107]. Hence it is not yet clear if Aβ oligomers are present in the original tissue, or rather arise due to experimental manipulations.

For example, SDS-PAGE can detect Aβ oligomers in human brain homogenates, yet surface-enhanced laser desorption/ionization time-of-flight MS (SELDI-TOF MS), which requires less manipulation of samples prior to analysis, fails to detect dimeric Aβ in human brain homogenates [107]. Both SDS-PAGE and SELDI-TOF MS could detect Aβ monomers from human brain homogenate. As the authors note, this suggest an over-reliance on low-resolution techniques, such as immunoblotting, may have distorted our understanding of Aβ biochemistry in the *in vivo* human brain. Clearly, our understanding of the true biochemistry and presence of Aβ monomers, dimers and higher order oligomers in the human brain *in vivo* is limited. Unfortunately, data that brought the amyloid hypothesis to its current, Aβ oligomer-based state, including the aspects concerning synaptotoxicity, arose from material driven by such low-resolution techniques.

#### Studying Aβ oligomer toxicity *in vivo* is methodologically difficult

As we have discussed above, support for a role of Aβ-oligomers in AD derives from experiments showing that injection of oligomers into the brain causes deficits in synaptic plasticity, learning and memory, and reduces synaptic spine density. However, there are several methodological aspects of these experiments which give cause for concern:

1. Some studies involve injection of synthetically derived peptides into the rodent brain, that were first crystallised into oligomers *in vitro*. These synthetically-derived peptides lack post-translation modifications, and may be different from Aβ peptides produced in the human brain [108].
2. In humans Aβ oligomers associate with lipoproteins, which may prevent Aβ-related toxicity, whereas synthetically derived Aβ are applied without these lipoproteins [109]. Clearly, this questions their physiological relevance.
3. Oligomerisation of Aβ may be stimulated by its adherence to the implanted plastic pumps used to deliver peptides [41].
4. The physiological relevance of injecting a bolus dose of either synthetically or human derived Aβ-oligomers into an intact rodent brain is doubtful, since this experimental protocol scarcely mimics the deposition of Aβ *in vivo* [110].

Clearly, models for testing Aβ oligomer toxicity *in vivo* must be considered in the context of our limited understanding of Aβ oligomer biology *in vivo* and in the context of technical limitations summarised above. Data from these studies cannot yet be confidently interpreted to elucidate the pathogenic mechanisms occurring in the human brain.

#### The role of Aβ oligomers in AD pathogenesis is uncertain

In sum, the doubts associated with Aβ plaques have driven the field towards considering oligomers as the central toxic species. However, a new set of problems arise from a lack of unequivocal evidence that Aβ oligomers are toxic *in vivo* [104]. There is yet to be a study convincingly establishing a relationship between specific conformations of oligomers and the initiation of disease *in vivo*, clearly in part due to methodological difficulties. Conflicting data from separate groups must be reconciled to gain a true understanding of Aβ biology in the normal and diseased brain. Standardising experimental protocols for identifying Aβ species is an important first step [111]. At this stage, a harsh interpretation is that perceived Aβ toxicity may represent experimental artefact rather than the true function of Aβ *in vivo* during the disease process. More likely, the role of Aβ, in whatever forms it is active, will surely ultimately need to fit into a broadened holistic view of disease.

## Key Observations in Human AD are Poorly Understood and Poorly Modelled

### AD symptoms and pathology are heterogeneous

The similarities between EOAD and LOAD pathology are integral to the amyloid hypothesis. Yet, both LOAD and familial EOAD are highly heterogeneous. They exhibit (1) different ages of onset [112], (2) differing temporal progressions [113], (3) different and varying cognitive symptoms [114] and (4) dissimilar pathological presentations [115].

Furthermore, a third of patients with EOAD show non-memory symptoms, whilst in LOAD only 6% have non-memory symptoms [116]. In twins environmental influences play a part in the timing of onset and on the levels of pathological markers at the end stage [117]. This heterogeneity has been recognised in recent AD diagnostic guidelines from the National Institute on Aging (NIA) and Alzheimer’s Association (AA) [118].

Important to note also is that although essentially 100% of individuals with Down’s syndrome have neuritic fibrillar plaques and NFTs by the fifth decade, the onset of dementia is highly variable, with only 70% becoming demented by their 70s, but with most maintaining their baseline cognitive abilities through their 40s and into older ages [13].

### AD brains show mixed pathological presentations

Regional aggregation of Aβ may differ in familial and sporadic cases. Remarkably, amyloid deposition is actually greater in some regions in sporadic AD cases than in early onset cases with *presenilin* mutations [119], indicating not all cases of AD follow the same distinct pattern of amyloid deposition as suggested in 2002 [120]. This observation alone raises doubt that the clinical phenotype of AD is solely related to Aβ deposition.

Furthermore, up to 50% of AD cases have mixed pathologies with other neurodegenerative conditions. For instance, α-synuclein deposition (otherwise seen in Lewy bodies), is a common co-morbidity with amyloid deposition, with more than 50% of AD patients also exhibiting α-synuclein accumulation [121].

Many consider disease heterogeneity as the manifestation of human genetic variation and environmental factors influencing progression of an Aβ or a tau-driven disease. Animal models provide support for this view, since they show homogeneous disease phenotypes, where variation can be introduced through environmental actions such as exercise and environmental enrichment and through mouse strain genetic background, providing a model for the amyloid hypothesis. However, as we shall next discuss, animal models may not truly reflect either LOAD, or even all cases of EOAD. A real possibility, instead, is that human AD may be heterogeneous in presentation because the causes of AD may be heterogeneous, causing the diversity of symptoms that characterise the disease.

### Pre-clinical AD models are not representative of human disease

Almost all mouse models of AD are engineered to over-express human APP to such an extreme extent that animals show pathology within months of birth. The treatments in current human testing have usually been shown to alter this pathology before being developed for the clinic. However, this approach has yet to produce a result that has translated to a positive human clinical outcome [66].

Little evidence indicates that APP is overexpressed in the human AD brain [122]. Indeed, total Aβ may be reduced [123]. More worrying, most mouse models do not show substantial neuronal loss, despite the presence of large depositions of amyloid [124]. Further, in contrast to human AD, where synapse loss is integral, mice show a highly variable presentation. Some mice show increased synaptic density in specific brain regions, while most models show reduced synaptic density.

Thus, while providing reasonable models for assessing the ability of a treatment to remove Aβ *in vivo*, and for investigating the relationship of Aβ to other features of the disease, such as its inflammatory components [125], the reality is that removing an overexpressed Aβ molecule in these mice may not be relevant to removing an under expressed, but aggregated molecule, from the human brain. Additionally, a number of questions remain as to the relevance of these transgenic mice to human AD:

1. Overexpression of wild-type APP, rather than mutant human APP, can cause memory impairments in mice independently of amyloid deposition [126,127]. APP overexpression may therefore only model rare forms of AD in which *APP* locus duplication is linked to EOAD [128]. Such duplication is associated with a very limited number of early-onset cases [129,130]. This questions the validity of mice overexpressing mutant APP. Further, it raises concerns about controls used in most mouse experiments; i.e. studies of age-related cognitive decline should use mice expressing wild-type APP at comparable levels to over-expressed mutant-APP, alongside the commonly used non-transgenic wild-types as controls, but usually do not [131].
2. Despite increases in amyloid, a number of mouse studies failed to detect any cognitive abnormalities [132]. For example, mouse models expressing familial AD-related mutant *APP* revealed no cognitive deficits [133]. Remarkably, in one report, overexpression of mutant human protein actually improved the cognitive performance relative to controls [134].
3. Mouse models of AD deposit peptides that are distinct from those found in the human brain, an important consideration in the design of drugs targeting Aβ removal [135,136].
4. The phenotype of AD mouse models varies depending on the background strain used, and can affect the outcome of drug studies [137]. Prudence therefore requires drugs to be investigated in multiple mouse lines and models, but this is not often done.
5. A related concern is that cognitive testing of mice requires updating [138].
6. While expressing the human *APP* gene, genetic animal models of AD also express endogenous, non-human APP. A critical question remains as to the role of endogenous mouse amyloid and APP in these models. Evidence suggests that the endogenous protein has an essential role in learning but this has barely been studied [139]. If true then removing endogenous Aβ in these mice, and indeed in humans, may have detrimental effects on memory, thereby contributing to the very problem they are designed to treat.
7. Both EOAD and LOAD are pathologically heterogeneous and many non-genetic risk factors for disease also exist, for instance Type II diabetes. Mouse models poorly represent these features of human AD.

### Development of novel pre-clinic models to improve translation of drugs to the clinic

Given the limitations of the mouse models, several groups have attempted to investigate alternatives. This includes other species that may better recapitulate AD pathology including rats, octodon degu, chicks, dogs, guinea pigs, rabbits, dolphins and non-human primates, although these are much more expensive to investigate and some still rely on APP over-expression.

Notable exceptions to the over-expressing mouse models include the senescence accelerated mouse model (SAMP8) [140] and the anti-NGF mouse [141]. These latter models replicate several features of AD without relying on human familial mutations. Others have used mice with inducible neuronal loss to replicate the patterns of loss seen in human AD [142]. Stimulation of inflammation also recapitulates many AD features in mice, including increased levels of cleaved APP fragments, altered tau phosphorylation [143] and declining motor and cognitive skills.

Recently a more relevant mouse model was created in which humanized Aβ, with human AD-causing mutations, was inserted into endogenous mouse *APP* [144]. These mice showed Aβ pathology, neuroinflammation and memory impairment, although there was an absence of tau pathology. This study supports a role for mutant *APP* (but not necessarily for Aβ *per se*) in some familial forms of disease. It does not however show Aβ causality in more common sporadic forms of disease. It is also prudent to recall that the majority of familial cases of AD are linked to mutations in *presenilin* genes, rather than mutations in *APP*, which are rare [145].

We are intrigued by the highly relevant modelling of AD based on other risk factors of disease. For example, diabetic mice develop many similar features to AD mice [146] and a mouse model of chronic heart failure shows alterations in the metabolism of cerebral Aβ and cognitive impairments [147]. These mouse models show it is not necessary to have familial AD mutations, nor do they need to have the aggregating form of amyloid, to re-create several features of disease. However in general, at this stage, the genetic mouse models hold front and center stage in AD studies. Studies based on these need to be increasingly treated with caution and consideration given to the use of different models.

## Genetics Paint a Complex Picture of AD Pathogenesis Beyond Aβ

### A complex picture indeed

It is widely accepted that Aβ, when injected or over-expressed in substantial excess, can cause pathology in rodents, but what is the scenario by which genetic mutations in the human cause AD? APP trafficking, function and cleavage is complex and highly controlled. We show in this section that mutations in *APP* can cause changes to a range of APP cleavage products, all of which could affect synaptic function. Meanwhile, *presenilin* mutations impact the cleavage of numerous proteins also with synaptic functions. Thus, even in EOAD caused by mutations in *APP* or *presenilin*, Aβ may not be the sole basis of disease. In sum, a valid, but inconvenient interpretation of AD genetics is that the aberrant processing of APP, or of other proteins cleaved by presenilin containing enzymes, is the key contributing factor in familial AD, rather than solely aberrant production of Aβ that contributes to histopathology. Furthermore, as we first discuss below, given the numerous genes and processes implicated in AD, it seems highly unlikely that any single gene such as APP alone will account for this disease in the majority of AD cases.

### The genetic risk factors for AD are many and varied

Genome-wide association studies (GWAS) have identified a number of genetic risk factors for AD. To date, the most emphatic demonstration that numerous genes contribute to the risk of AD has come from a meta-analysis of four GWAS data sets consisting of 17,008 AD cases and 37,154 controls from 15 countries [148], which implicated 11 new regions of the genome as risk factors for AD. The findings reinforce the importance of the innate immune response and inflammation (*HLA-DRB5/DRB1, INPP5D, MEF2C*) already implied by previous work (*CR1, TREM2*). Also reinforced is the importance of cell migration (*PTK2B*), lipid transport and endocytosis (*SORL1*). New hypotheses on AD pathogenesis have also emerged related to genetic mutations in molecules associated with hippocampal synapse function (*MEF2C, PTK2B*), the cytoskeleton and axonal transport (*CELF1, NME8, CASS4*) as well as myeloid and microglial cell functions (*INPP5D*). While efforts are often made to relate new data to the amyloid hypothesis [149], many of these mutations are not conveniently placed within it.

Furthermore, new research showing the molecular signatures of AD vs. normal aging indicates that the molecular phenotype of AD is highly complex, with a variety of transcriptional changes differentiating AD from aging. Transcriptional profiles for neuroinflammatory and lipid metabolism genes in particular are altered early in disease in this dataset [150].

GWAS and ageing data is therefore increasingly consistent with a view that Aβ (or more likely altered APP production, function and cleavage) exists somewhere within a highly complex disease framework that is yet to be understood. It is unclear whether numerous mechanisms converge on a single primary pathway, or, if AD will need to be redefined as a host of diseases manifesting ultimately as memory loss, resulting from synapse loss and neurodegeneration. The latter view, that memory becomes problematic when brain function is disrupted, has simple appeal, but is a nightmare from a therapeutic perspective.

### Mutations in *presenilin* genes do not always increase Aβ cleavage

There is a widespread assumption that all the genetic links to AD effectively modify the cleavage of Aβ to produce more of the longer forms, Aβ42 and Aβ43, or increase the ratio of longer Aβ peptides compared to shorter ones and in turn that this is causative of AD. Certainly, evidence for increased levels of Aβ42, or for increases in the Aβ42:Aβ40 ratio as a result of *APP*, *PSEN1* and *PSEN2* mutations has been found in both *in vivo* and *in vitro* studies [151-153]. However the conclusion is not warranted in view of the full data set.

In a study examining the effects of eight FAD *PSEN1* mutations on Aβ production, most of the mutants produce no change in the Aβ42:40 ratio [154]. Furthermore, family members with the same FAD mutations exhibit heterogeneity in their clinical and neuropathological phenotypes [155]. These results are supported by studies showing heterogeneous effects of FAD *PSEN* mutations on the Aβ42:40 ratio depending on the mutation [156-158], but are contradicted by reports of universal increases in Aβ42 as a result of FAD mutations [153,159]. Nevertheless as it stands, it seems unlikely that FAD mutations lead to the same phenotypic amyloid cleavage, resulting in increased Aβ42 and/or increases in the Aβ42:40 ratio.

Newer evidence for the role of a longer form of Aβ, Aβ43, in disease pathogenesis may be the result of a familial AD-linked *presenilin* mutation [160]. The Aβ43:Aβ42 ratio is increased in mice harbouring this particular *presenilin* mutation, with no change in Aβ40 or Aβ42 levels [160]. We simply cannot draw conclusions at this stage about the role of any form of Aβ in disease. It would be prudent to include measurements of a variety of Aβ cleavage forms in disease, and determine the importance of qualitative versus quantitative changes in Aβ production over time, during disease [161].

### Presenilin has important physiological functions independent of Aβ cleavage

Evidence from rare clinical case studies illustrates mutations in *presenilin* genes can be associated with neurodegeneration independently of amyloid plaque deposition. *Presenilin* mutations have been found in frontotemporal lobe dementia (FTD) without amyloid pathology [162], dementia with Lewy bodies [163], posterior cortical atrophy dementia [164] and atypical dementia [165]. However newer evidence suggests *presenilin* mutations may not be the true causes of all these amyloid-independent neurodegenerative states, as genetic defects in the *progranulin* (*PGRN*) gene can explain FTD, atypical phenotypes and parkinsonism, also associated with *presenilin* mutations [166,167]. There is however at least one clinical case involving a point mutation in *PSEN1*, that is associated with the development of Pick’s disease with tauopathy, but without amyloid plaques [168].

Regardless, in AD, where amyloid plaques are present, there is good reason to suggest that the effects of *presenilin* mutations on APP biology may account only in part, if at all, for the AD pathology they associate with. In fact, there are several known functions for presenilin which could be impacted by FAD-linked *presenilin* mutations. These alternative functions include roles in macroautophagy, APP vesicle transport, cell survival, cleavage of a wide variety of possible substrates, and the importance of presenilin for synaptic function, which together have culminated in the presenilin hypothesis of AD [169]. There are also links between presenilin function and the innate immune system, which may account for the presence of neuroinflammation in EOAD, independently of amyloid [170]. The numerous effects FAD-linked *presenilin* mutations would have on all these processes could together account for AD independently of, or additional to, any effect on amyloid pathology.

### The Presenilin hypothesis of AD

A ‘presenilin hypothesis’ of AD has been articulated [169]. We further suggest that *presenilin* mutations fit best within a hypothesis that AD is a disease driven by synapse loss. Though the AD literature has largely focused on the role of PSEN1 in APP cleavage (Figure 2), *presenilin* mutations affect a range of proteins and therefore processes, particularly those involved in synaptic function, as summarised above. Given that presenilin appears important for cleaving proteins that are crucial at synapses, *presenilin* mutations would lead to synaptic dysfunction. It would also follow that drugs targeting presenilin in humans are destined to have profound detrimental effects on the brain with long-term use. This indeed was the result of recent clinical trials of presenilin antagonists, also termed γ-secretase inhibitors (Table 1).

Nevertheless γ-secretase inhibition continues to be pursued. Recent evidence using conditional *presenilin* KOs has suggested that presenilin function is not as critical in the adult brain as the developing brain, allowing the field to justify its ongoing use as a target for drug intervention (See [171] for a detailed review). This is in spite of the reservations that emerge from consideration of data reviewed above.

### APOE4 dysfunction is related to inflammation

The *APOE4* allele has been a known genetic risk factor for sporadic AD, and it remains the strongest known [22]. It has been linked to the amyloid hypothesis by indications it is involved in the clearance pathway of Aβ, with deficits causing a toxic Aβ accumulation and aggregation [172]. Meanwhile, an alternative avenue of enquiry shows that APOE4 has intimate connections with innate immunity, and this was reasoned to explain its broader relationship with inflammatory disease, not just AD. One observation is that APOE suppresses TNF secretion from inflammatory cells [173] (also see [174]). Importantly an APOE mimetic that suppresses TNF secretion has successfully treated experimental models of neurodegenerative disease, including traumatic brain injury [175], stroke [176] and AD [177]. As well as reducing behavioral deficits, in the study of AD, the APOE mimetic also reduced Aβ plaques and tau tangles [177]. The relationship of APOE4 to inflammation therefore opens a channel of enquiry directed to explain why stimulation of APOE expression in mice enhances normal Aβ clearance (both soluble oligomers and plaques) and reverses behavioural deficits. In line with the observation of the link between *presenilin* mutations and inflammation, the links between APOE4 and inflammation further point to inflammation as a major player in AD pathogenesis independently of Aβ.

### Understanding the complexity of APP biology independently of Aβ is important to understanding AD pathogenesis

APP synthesis, trafficking and cleavage are complex and highly regulated processes (Figure 2). It is important to recognise that familial AD *APP* and *presenilin* mutations may not only impact Aβ production, but also the production of the other peptides produced from APP including sAPPα, sAPPβ, p3 and AICD, as well as the relative levels of full-length APP. Interestingly, overexpression of AICD can cause an AD-like phenotype [178], whilst increased cleavage of sAPPβ is associated with familial Danish Dementia with similar aetiology to AD [179]. Furthermore lowered levels of neurotrophic sAPPα are seen in AD, and mutations which inhibit the α-secretase enzyme ADAM10, which liberates sAPPα from its precursor, are found in the promoter region and coding sequence of some individuals with AD [180]. Depletion of sAPPα by inhibition of ADAM10 trafficking can bring about sporadic AD phenotypes [181], corroborating an independent role for APP cleavage products other than Aβ in bringing about disease phenotypes.

Whilst the functions of p3 and sAPPβ are little explored (which in itself is a remarkable reflection of the intense focus on Aβ at the expense of other cleavage products of APP), a wealth of evidence exists for physiological functions of sAPPα, Aβ, AICD and full-length APP [182] (Figure 2). These studies raise questions as to whether familial AD driven by *presenilin* and *APP* mutations is primarily a result of aberrant Aβ expression, or if it is in fact a result of altered APP cleavage, and the resultant effects of altered APP cleavage on sAPPα, sAPPβ, Aβ, AICD, p3 and full-length APP. This brief discussion does not even approach the possible physiological functions of C83, C99 (Figure 2), the different functions of the multiple isoforms of APP including APP_695,_ APP_751_ and APP_770,_ or the highly homologous proteins to APP, APLP1 and APLP2, which are also physiologically expressed in the human brain and may serve redundant functions with APP proteins.

### There are many unresolved issues in the amyloid hypothesis

The field has pursued the idea that Aβ accumulation is the central cause of AD based in part on an amyloid-centric interpretation of the genetics. Yet as described above, numerous products of APP are affected by *APP* mutations and by *presenilin* mutations.

One valid conclusion, in view of the complex biology of APP function and cleavage, is that *APP* and *presenilin* mutations cause AD because they alter several cleavage products of APP, which each in turn contribute to AD. In this view of disease, alterations in Aβ expression would only be a part player in pathology or, perhaps even, a by-product, and an indicator of altered APP function and cleavage. This conclusion, if true, would predict that clinical trials of anti-Aβ drugs will fail even in some or all cases of EOAD.

Meanwhile, analysis of the effects of *presenilin* mutations does not lead to the conclusion that AD is caused by Aβ. The cleavage of a range of proteins is affected by *presenilin* mutations and many would affect the synapse. In fact as we illustrated, *presenilin* mutations do not always alter Aβ production as may be expected. Finally, many mutations and risk factors associated with AD may not relate to Aβ metabolism.

Yet, amyloid-centric interpretations continue to flourish. A recent study showed that a mutation, determined to modestly decrease Aβ levels of the course of lifespan, is preventative of AD [23]. This data was taken to suggest that a life-long reduction in Aβ reduces the risk of AD. However, the mutation also results in marginally (albeit non-significant) increases in levels of beneficial neurotrophic sAPPα. Regardless, an alternative interpretation is that a life-long change in APP function and cleavage could protect against AD independently of lowered Aβ production.

## Future Directions

### The emergence of more holistic approaches to understanding AD pathogenesis

**Figure 3:**
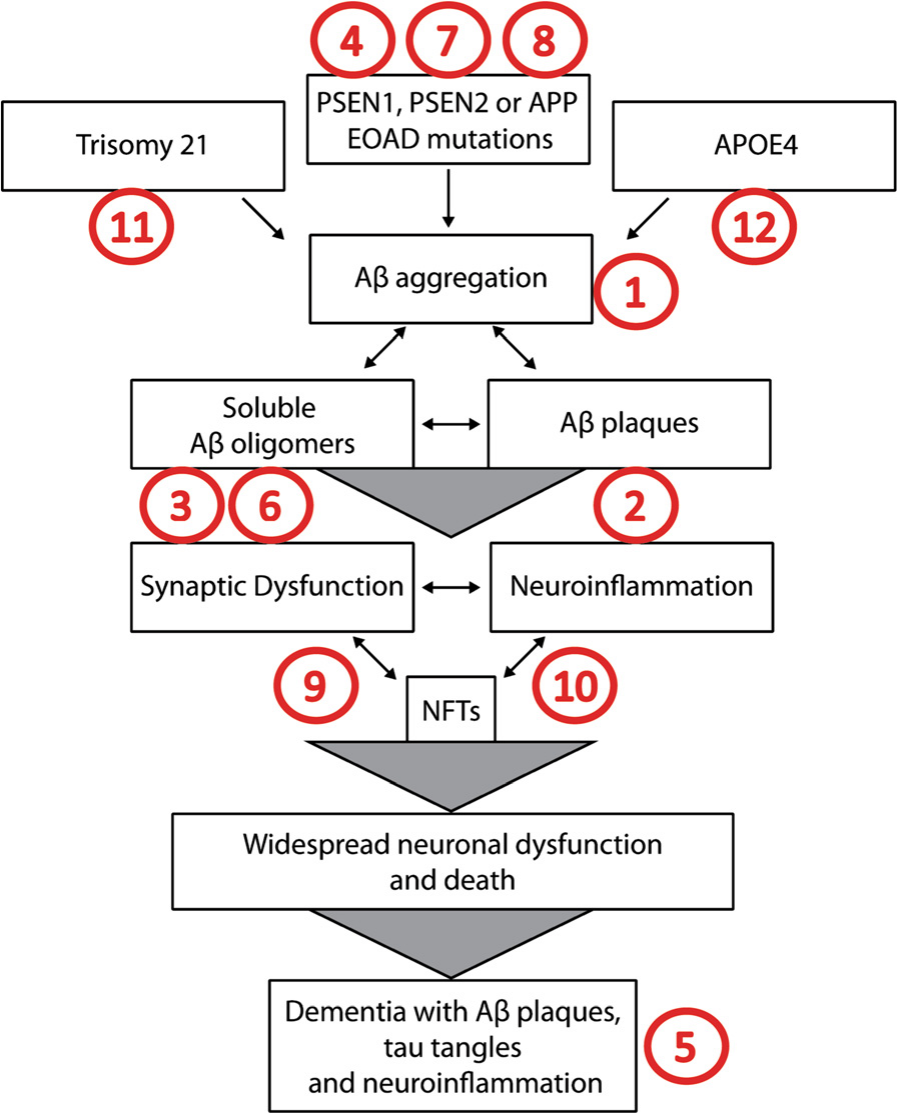
**Controversies and Inconsistencies Within the Current Amyloid Hypothesis. 1.** Aβ deposition occurs in cognitively normal individuals; **2.** There is a weak correlation between plaque load and cognition; **3.** The biochemical nature and presence of Aβ oligomeric assemblies *in vivo* is unclear; **4.** Pre-clinical AD models based on EOAD-linked mutations are biased toward the amyloid hypothesis; **5.** Pathological heterogeneity and comorbidities are unexplained by the amyloid hypothesis; **6.** Aβ has a normal physiological role and targeting Aβ may disrupt these roles over the long term; **7.** Genetic factors linked to AD can be interpreted independently of amyloid; **8.** APP cleavage and function is more complex than solely the production of Aβ, indicating other APP family members may play a role in disease progression; **9.** The triggers of synapse loss, neuronal loss and neuroinflammation in AD are still unclear; **10.** The relationship between Aβ and tau pathologies is unclear; **11.** The onset of dementia in Down’s Syndrome is highly variable, despite the presence of fibrillar plaques in 100% of Down’s individuals by the fifth decade; **12.** The *APOE4* genotype has numerous functional effects, rather than solely relating to reduced Aβ clearance, including links to enhanced inflammatory phenotypes. Each of these points are discussed in detail in the text.

As suggested by Figure 3, the amyloid hypothesis is at least incomplete, and quite possibly largely incorrect. Therefore it follows that therapies targeting Aβ or APP processing may not treat LOAD, and possibly may not even work in some cases of EOAD. Given this conclusion, it is worthwhile to consider alternative possibilities. There are a number of theories in the literature that must be given serious consideration and ultimately integrated into a holistic view of disease. We will elaborate on just some of these, below.

### Insulin resistance and Inflammation

It is suggested that a similar pathogenesis operates in AD as in Type 2 diabetes (T2D), but restricted to the brain, thus describing AD primarily as a result of cerebral insulin resistance [183]. Certainly cerebral insulin resistance is present in AD just as in T2D [184], and the appropriate alterations in post-insulin receptor intracellular signalling have been impressively demonstrated in fresh AD autopsy brains [185]. This idea is likely inseparable from the argument that AD is an inflammatory disease, since evidence that excessive TNF induces insulin resistance is biochemically precise [186] and, as has been reviewed [187], is widespread across many inflammatory diseases, infectious and sterile. Moreover an agent that inhibits TNF production [188] and another that controls insulin resistance [189] have both been shown to reverse AD in experimental models [190].

### The inflammatory hypothesis of AD is a valid alternative to the amyloid hypothesis

We and others have long proposed a role for neuroinflammation, driven by microglia and astrocytes, as a trigger for Alzheimer’s pathogenesis [46,125,187,191]. The case for chronic inflammation, as classically defined, rather than Aβ, being the primary initiator of AD has a long history, with new evidence continuing to accumulate. From 1989 it has been reported that inflammatory cytokines are essential for the excess APP required for the amyloid hypothesis of AD [192], as well as up-regulating its cleavage to form Aβ [187]. In addition, parallel studies demonstrating that oligomeric Aβ influences synapses through inducing the inflammatory cytokine TNF [187,193,194] have been enlightening. A possible role for neuroinflammation in synapse pathology early in disease has now been acknowledged [195] and there is much evidence, from genetics and measuring indicatory of inflammation very early in AD, that it is in the right place at the right time to be causal, and likely to precede Aβ and tau pathologies [187]. More recently, the clinically approved specific anti-TNF agent, etanercept, is reported to prevent changes caused by administering Aβ to mice intracerebroventricularly [196].

### The tau hypothesis of AD

The concept of hyperphosphorylated tau being a primary mediator of AD, like amyloid, has a long history, which continues to grow [197]. Much interest still exists in where tau sits in the pathogenesis of AD [198]. In our view, AD is sufficiently diverse that it is conceivable that the role of tau, and where it sits in AD pathology, could vary among individuals. If tau is a primary activator of disease in some cases, it is imperative that the reported harmlessness of phosphorylated tau to neurons during mammalian hibernation [199] be discussed in AD research circles. Furthermore, hyperphosphorylated tau can be considered another histological sign of cytokine activity [187].

### Redefining ‘neuroinflammation’ through viewing the synapse as a complex multicellular structure is important in future AD research

The inflammatory hypothesis is an example of how amyloid and tau research can be integrated into a novel set of ideas, both expanding the amyloid hypothesis and including it. However, while we use the term ‘neuroinflammation’ throughout this text and elsewhere, we note that neuroinflammation is poorly defined. In its simplest form neuroinflammation is currently defined by altered glial cell morphology and excess pro-inflammatory cytokine release [46]. This must ultimately give way to a more complex and subtle view of glial function/dysfunction within the multicellular synapse [200].

Stepping back to consider the multitude of factors we have summarised above, a complex picture emerges that consistently points to synaptic dysfunction and loss as a major link between the diverse characteristics of the disease. We have recently pointed out the synapse needs to be re-defined and understood as a multicellular structure where glia play a critical role [46]. This allows us in turn to re-imagine AD.

Microglia and astrocytes are essential to normal synapse biology, including the removal [201,202] and formation of synapses [203,204], and maintenance of synaptic function [205,206]. Disruptions in signalling between glia and synapses, which may involve several known cytokines such as TNF, could therefore drive the well-known synapse loss in AD, either independently of, or in conjunction with Aβ [46].

A consequence of this interpretation is that the issue may not be an upregulation of neuroinflammatory signalling from these cells *per se,* that is involved in disease. Rather, expression of pro-inflammatory cytokines and other neuronal and glial derived molecules regulating the synapse could be disrupted subtly for a host of reasons, well before frank inflammation is apparent. APP, along with presenilin and indeed numerous other factors, may exert effects on the synapse through actions on glial function, leading to either excess synapses (as occurs in autism) or synapse loss (as occurs in AD). This would modify glial function at synapses, and potentially drive synapse loss. Thus, we propose that many of the factors thought to cause inflammation are more likely to cause a dysregulation of glial function at the synapse in the first instance, long before changes in cell morphology become obvious. Consequently, more subtle mechanisms may underpin AD.

Clearly, understanding the physiological roles of microglia and astrocytes at synapses, as opposed to simply considering them as cells with key roles in innate immunity and ‘neuroinflammation’ , is a critical avenue for future research. We suggest future research will reveal that the entire current concept of ‘neuroinflammation’ is poorly understood, defined and characterised. This concept will require profound rethinking before we can truly understand the role of glia in the unperturbed brain and in AD pathogenesis [46].

## Conclusion

In the words of Joseph Lister (1876)

*"In investigating nature you will do well to bear ever in mind that in every question there is the truth, whatever our notions may be. This seems, perhaps, a very simple consideration, yet it is strange how often it seems to be disregarded. I remember at an early period of my own life showing to a man of high reputation as a teacher some matters which I happened to have observed. And I was very much struck and grieved to find that, while all the facts lay equally clear before him, those only which squared with his previous theories seemed to affect his organs of vision."*

Lister’s quote is salient. Hypotheses are an important part of any scientific method, but the sentiment of Karl Popper, quoted earlier in this article, should be taken seriously. Keeping Popper’s views in mind may prevent us from becoming over-reliant upon, and blinkered by, any single hypothesis for AD.

It has been said the amyloid hypothesis, like certain banks, may have become too big to fail [101]. The hypothesis may yet prove its merit, at least in some cases, through early intervention trials with amyloid-directed therapeutics [70,71]. However, on the basis of the data discussed here, the role of Aβ as a primary cause of all AD remains debatable. We are therefore concerned by the suggestion that, if anti-Aβ treatments are successful in patients with EOAD, this would support an argument for treating all AD with anti-Aβ drugs [207]. Such a conclusion would merit questioning without direct clinical evidence that the treatments are effective in LOAD.

We are not arguing that Aβ has no role. In fact it may be a player in a more complex view of disease and, further, its role may even be variable. We suggest instead that to solve the complex riddle of AD, theoretical models must expand beyond Aβ as the central cause of dysfunction, instead including Aβ in a wider theory that accounts for the extensive data and advances in neuroscience that have accumulated over the last decade. Ultimately it is critical that any role for Aβ must be placed in the context of a holistic view of the disease that accounts for all the data.

Even more so, with recent meta-analyses highlighting some major pitfalls with experimental design and statistical power in neuroscience [208,209], we need to be wary. Conclusions drawn from any experiment must be replicated before accepting them as fact, especially considering the difficulty in replicating *in vivo* studies when using different background animal strains, and different methodological approaches [210,211].

An important suggestion we make is that the concept of neuroinflammation mediated by glia may need to give way to a more subtle understanding of how aberrant glial function at synapses drives AD. We suggest an alternative view that, given evidence for synapse dysfunction as an early event in AD, synapse dysfunction may *ipso facto* be the cause of AD. We recently suggested [46] a new definition of the synapse as “*…a complex, dynamic and often transient structure involving several cells interacting within a sophisticated extracellular matrix and milieu*.” Within this framework, one of the normal roles of glia in synapse structural plasticity is to modulate and also remove synapses. Improving our understanding of how dysregulation of the multicellular synapse leads to aberrant synapse elimination will likely produce novel insights into mechanisms of synaptic degeneration in AD, and provide insights into the relationship between synaptic degeneration and other pathological hallmarks of the disease. The corollary of this is that if we can identify signaling pathways that reverse glial mechanisms leading to synapse removal, we may identify approaches that could halt or even reverse AD, independent of specific cause. Regardless, if synapse loss is one of the earliest events in disease then we must go back to first principles and understand what drives this loss.

The primary point of our review is to suggest it is inappropriate to ignore equally valid interpretations of data. There are many thousands of papers on Alzheimer’s disease, and many of these papers can be interpreted in alternative ways, while still more are contradictory to, and/or inconsistent with, the amyloid hypothesis. There are also many thousands more investigating mechanisms driving synapse function and dysfunction that could be linked to AD literature, given synapse dysfunction is a key early event and accurate correlate of AD progression. We conclude by suggesting the students, post-docs and young faculty who will determine the course of AD research in the next decade, must spend time reading this literature extensively, and thinking deeply, and thus become the next generation of leaders that, at the expense of time away from the lab bench, determine the best pathway forward.
